# Practical model for workforce development and community engagement in the public health laboratory

**DOI:** 10.3389/frhs.2026.1819238

**Published:** 2026-06-16

**Authors:** Aubrey L. Galusha, Brooke Clemons, Brittany O'Brien, Abigail Smith, Nicholas Jones, Lindsay Toffolo, Michele Kilmartin, Lisa Biega, Quillan Brady, Alexander C. Keyel, Fida Kased, Kara Phipps, Spencer A. Bruce, Leonard F. Peruski

**Affiliations:** Wadsworth Center, New York State Department of Health, Albany, NY, United States

**Keywords:** community engagement, cross-disciplinary collaboration, employee engagement, organizational change, public health laboratory, workforce development

## Abstract

**Introduction:**

Numerous reports detail the challenges facing the public health laboratory workforce. We focused on an action-oriented solution, scaled to the largest state public health laboratory in the US: the Wadsworth Center of the New York State Department of Health. Here, we report findings from four activities developed/supported by the Community. Advancement. Recruitment. Engagement. (CARE) initiative.

**Methods:**

Over 18 months, CARE re-established academic engagement activities, supported the formation of learning groups, assisted the development of an Orientation Program, and established a formal Steering Committee.

**Results:**

Programmatic success based on quantitative outcomes was clear: >1,000 students reached through academic outreach, 79% of Orientation Program participants correctly responded to all assessment questions, 35x higher engagement with learning modules from participants in a learning group, and 13 objectives completed in the initial Steering Committee term. In addition, participant satisfaction was ≥70% for all activities.

**Discussion:**

Challenges included dedicated time/logistics, minimal budget, and navigating formal process/procedure. CARE's success in developing cross-center relational infrastructure and engaging staff in meaningful opportunities to contribute has established a strong foundation for future activities. Our work provides a realistic, low budget, integrated model, deployable in scientific and diverse public health institutions.

## 1 Introduction

Rapid advances in laboratory instrumentation, automation, and computational technologies have created unprecedented opportunities to strengthen the prevention, detection, and response to public health threats. At the same time, public health laboratories face an increasingly fragile workforce landscape. In the decade preceding the COVID-19 pandemic, public health agencies experienced sustained reductions in full-time equivalent staff despite population growth, along with persistent challenges in recruitment, training, and retention (1).

The COVID-19 pandemic intensified these workforce pressures. Emergency mass hiring was necessary to meet testing and response demands but introduced new challenges related to onboarding, integration, and training (2). In parallel, the prolonged intensity of pandemic response had substantial effects on workforce well-being; one study reported that nearly 25% of public health agency staff experienced at least three symptoms of post-traumatic stress (3). The cumulative impact of these trends has left many public health laboratories confronting ongoing challenges related to recruitment, staff satisfaction, professional development, engagement, and retention.

In response to these challenges, the Wadsworth Center launched the Community. Advancement. Recruitment. Engagement. (CARE) initiative in early 2024 as a practical, action-oriented framework for institutional change. CARE was designed to address structural and cultural barriers to workforce sustainability while strengthening the Center's ability to fulfill its public health mission. Through an initial assessment, the CARE team identified three strategic priority gaps essential to scientific innovation and effective public health practice:

1. Effective collaboration within the profession,
2. High technical competence, and
3. Resilient connections with the communities served.

Concurrent evaluation activities, including a series of structured “Listening Sessions,” assessed baseline staff perspectives related to these priority gaps. These sessions identified communication, connectedness across the Center, limited opportunities for professional development and growth, and insufficient visibility within the broader community as four of the six most critical workforce challenges.

CARE was intentionally structured to facilitate effective collaboration through the development of trust, a shared purpose and vision, and collective confidence in the team's ability to achieve its goals – factors consistently associated with high-performing teams (4–7). During its first 18 months, CARE piloted four targeted initiatives designed to enhance technical competence, strengthen community relationships, and establish sustainable structures for long-term institutional impact:

1. Academic outreach to local schools to inspire interest in science and public health while strengthening community ties;
2. A comprehensive Wadsworth Center Orientation Program to ensure new staff understand the Center's mission, resources, and culture;
3. Learning groups that support community-based training, shared skill-building, and peer-to-peer professional development; and
4. Establishment of a program steering committee to guide implementation, maintain momentum, and support sustainability.

Here, we describe the CARE initiative's early successes, challenges, and future directions, with the goal of providing a practical, scalable model for workforce engagement and development that can be adapted across diverse public health and scientific institutions.

## 2 Methods

### 2.1 Institutional description

The Wadsworth Center is the largest state public health laboratory in the United States and currently employs approximately 800 staff across scientific, support, and trainee roles – staff are funded through state and federal resources, and are represented by multiple unions. Most staff are assigned to one of five primary physical sites in Albany, NY, with additional staff in other regions of New York State. Organizationally, Wadsworth Center includes a director's office and seven divisions: infectious disease, genetics, environmental health sciences, scientific cores, translational medicine, laboratory quality certification, and laboratory operations.

### 2.2 Academic engagement

The CARE program identified a need within the Albany area for scientists to connect with high school students. In response, a database of teachers and counselors in the New York Capital District was created to support sustained collaboration and facilitate programming. Wadsworth Center staff reached out to 92 contacts across 17 high schools to offer opportunities for classroom visits, lab demonstrations, and career pathway discussions, and collaborated with respondents to customize experiences based on curriculum goals, student interests, and the Center's expertise/capacity. Internally, CARE worked to match staff with opportunities, supported them, and organized several larger events. In addition, CARE facilitated external requests for tours and set up a pilot process and tracking system for academic engagement more broadly.

### 2.3 Wadsworth Center orientation program

The Wadsworth Center Orientation Program is a staff-led initiative developed by a group of individuals who were enabled and supported by CARE. Orientation Program goals and specific learning objectives were developed to address institution-specific gaps in the onboarding process. The orientations were scheduled for one full workday and included presentations by each division, a facility tour, demonstration of resources, and activities to facilitate staff connections.

### 2.4 Learning groups

Four Udemy-based learning groups were established to promote professional development in both technical and leadership domains. Udemy is an online learning platform with license-based subscriptions to >30,000 courses and is one of the resources available to staff within the Wadsworth Center. The groups focused on artificial intelligence, Python, R, and leadership/management based on structured discussions with staff and availability for an experienced group lead. Each group provided structured opportunities for participants to strengthen skills highly relevant to Wadsworth Center's current and future needs. A summary of the groups, including membership and key activities, is provided in Table 1.

**Table 1:** Description of the four learning groups.

Topic	Size	Frequency	Structure
Artificial Intelligence	14	Monthly	Multi-level group working through beginner-to-advanced material discussing applications
Leadership	9	Biweekly	Selected courses to complete together and discussed successes and challenges in application
Python	5	Biweekly	Used one Udemy class to learn then focused on using Python for lab workflow
R	13	Biweekly	Open format; roundtable on progress, space for questions, and highlights of use cases

The learning groups were fully operational from October 1 through December 31, 2024. Notably, three of the four groups elected to continue meeting beyond the pilot period, reflecting strong participant engagement and perceived value.

### 2.5 CARE steering committee

The CARE Steering Committee was adapted from an established Workforce Champions initiative within the Centers for Disease Control and Prevention (8). Significant efforts were made to facilitate a representative Steering Committee that reflected the target audience of the CARE Program. Nominees for the inaugural Steering Committee were solicited in Spring 2024 from leadership across the Center and selected following a brief interview process.

The Steering Committee launched September 1, 2024 with 24 members from all five campuses, representing six of the seven divisions that comprised the Wadsworth Center at that time, as well as the Director's Office, the Information Technology Group, Administration, and trainees. The Committee was organized with a Chair overseeing five groups, each group with a designated lead and liaison: academic engagement, community engagement, internal community engagement, recruitment, and training/professional development.

### 2.6 Initiative evaluation

Table 2 shows an individualized evaluation framework for each of the four activities described above. Each was evaluated with a combination of quantitative metrics and participant feedback to assess specific goals, gather feedback from participants, and evaluate impact on applicable overarching goals:

1. Build connections across staff within the Wadsworth Center.
2. Engage staff to develop themselves and contribute to the Center.
3. Establish visibility within local schools as the State Public Health Laboratory.

**Table 2:** Evaluation framework for CARE pilot initiatives.

Initiative/Activity	Primary Workforce Gap(s)	Quantitative Indicators	Qualitative Feedback	Short-Term Outcome(s)	Long-Term Expected Outcome(s)
Academic Engagement	Limited visibility of public health laboratories	# Students # Schools/programs # Staff participants	Staff feedback surveys	Establish feasibility of formalized academic engagement for outreach.	Build relationships with the community, inspire the next generations of scientists.
Orientation Program	No Center-wide system in place to integrate new staff. Confusion among staff and supervisors about where to go for accurate information. Lack of understanding for the scope of the Center and how their role fits.	Attendance % assessment questions correct	Participant feedback surveys	Orientation attendees know where to find resources, can relate how their role fits with the Center's mission, and meet individuals outside their immediate group.	Strong connection between staff roles and the organizational mission.
Udemy Learning Groups	Limited usage of developmental tools available for staff Limited development of programming and leadership skills	Time spent on the platform	Participant feedback surveys	Licenses are actively used	Application of learned skills to workplace.
Steering Committee	Few opportunities for staff at all levels to contribute to their workplace	# activities completed/in-progress. # staff engaged in activities. Variety of activities	Committee feedback surveys	Staff are enabled to act	CARE remains sustainable, adaptable, and broadly representative of the institution

Surveys were administered via Microsoft Forms for academic engagement, learning groups, and the Steering Committee. The Orientation Program survey was given during a dedicated time slot during the session. All feedback was collected anonymously. Qualitative responses were grouped thematically based on consensus among at least two individuals. Numerical responses were analyzed and visualized using R (R Foundation for Statistical Computing, Vienna, Austria), version 4.5.0. Data were reshaped and summarized using the tidyverse suite of packages, and figures were generated using ggplot2.

## 3 Results

### 3.1 Academic engagement

Over 16 months, 88 staff at the Wadsworth Center were involved in 28 events that included activities such as laboratory tours, demonstrations, classroom presentations, career fairs, and experiential learning opportunities. In total, approximately 1,030 students interacted with staff through 21 school districts/programs. Three school districts reached out to us for additional opportunities following an initial event. The number of students in each event ranged from 2 to ∼140 and the primary audience was high school students (∼840).

**Figure 1:**
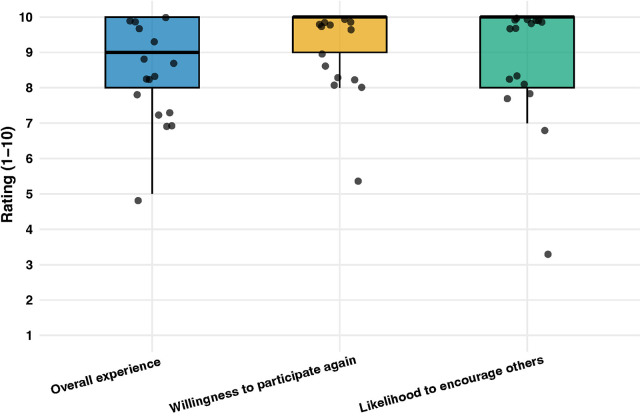
Numerical responses to survey questions completed by staff that took part in at least one academic engagement activity.

Survey results are shown in Table 3 and Figure 1 from 23 staff who participated in at least one academic engagement event for open text questions and numeric response questions, respectively. Overall feedback indicated high satisfaction with participation – > 95% of staff rated their overall satisfaction of the event ≥7/10, responded that they would participate again, and recommend others to participate in a future event. Staff indicated enjoyment from working with students, discussing science, and meeting others from the Center, and 15/20 responses noted they learned or developed at least one skill.

**Table 3:** Thematic grouping of open-text responses to survey questions completed by staff that took part in at least one academic engagement activity.

Response	Number
What did you like most about the event?	
Working with kids	10
Talking about science	8
Meeting colleagues	4
Other (giving back, working with school staff, and hand on activities)	5
What was the least satisfying aspect of the event?	
Preparedness/organization	6
Length/timing of event	5
Internal policies and procedures	3
Other (nothing, coordination with staff, WC support, etc.)	9
Did participation in the event help you learn or develop any skills?	
Public speaking/presentation skills	7
None	5
Learning more about Wadsworth and collaboration	4
Other (time management, working with kids, communicating science)	4
In the future, what would have do differently?	
Be more prepared	11
More hands-on activities	4
Shorten presentation	4
Other (none)	4
Do you have any ideas for future academic outreach events?	
None	14
Reach more schools	7
After-school programs	4
Other (science fair judging)	2
Do you have any additional feedback?	
Thank you and keep up the good work	3
Appreciate access to outreach material/resources	1
Funding for events	1
Other	1

The most common difficulties for participating staff were preparation and organization, event timing, and navigating policies and procedures. Other lessons learned included shortening their presentations and using more hands-on activities for the students. Staff suggested targeting after-school activities and clubs, career fairs, and science fair judging, and would like to see appreciation for their work on academic outreach, improved access to outreach materials and resources, and funding for the program.

### 3.2 Wadsworth Center orientation program

**Figure 2:**
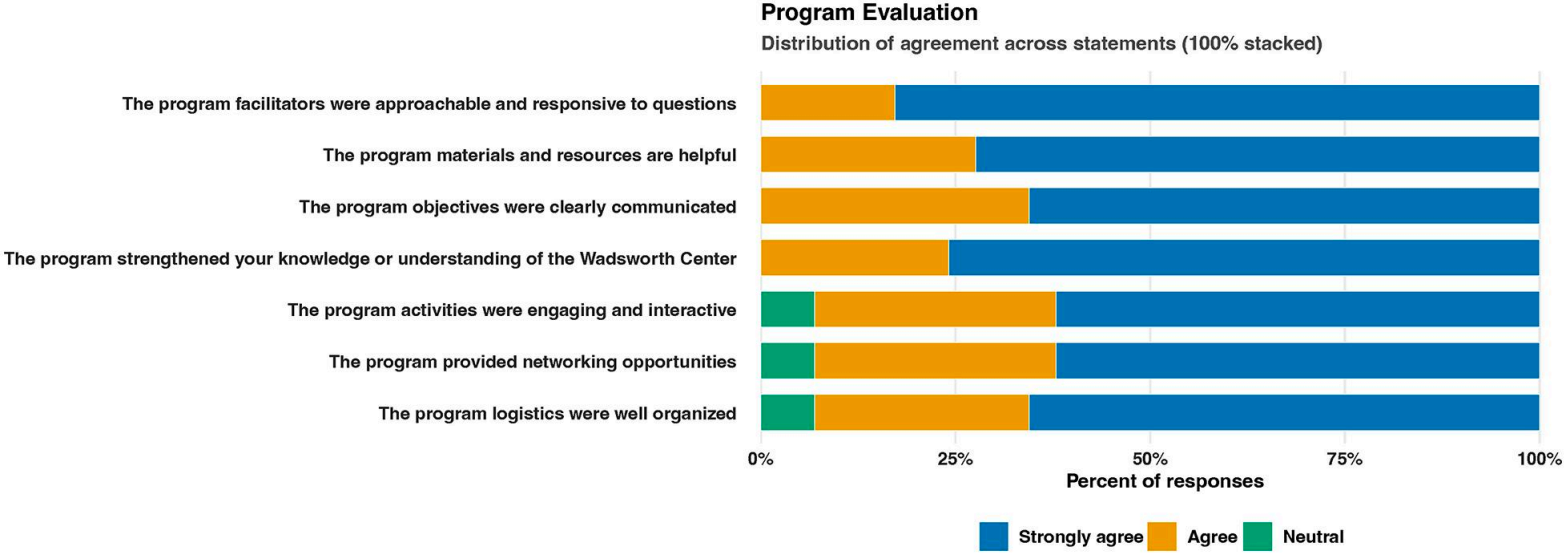
Likert summary of agreement for orientation program evaluation.

Evaluations from 29 to 32 (91%) attendees from the initial two Wadsworth Center Orientation Program days are summarized in Figure 2 for seven Likert questions and Table 4 for open-text feedback from the initial event. Over 85% of respondents answered “strongly agree” or “agree” to the program rating questions in Figure 2. In total, 79% of attendees correctly answered questions directed at assessing learning objectives:

1. Summarize the Wadsworth Center mission statement.
2. Describe how their new role fits into the Center's mission.
3. Identify the different Wadsworth Center divisions.
4. Name three coworkers outside of the new employee's immediate work group and briefly describe their roles.
5. Access important employee resources.

**Table 4:** Thematic grouping of open-text responses to survey questions completed by staff that took part in the initial orientation day.

Response	Number
What was the most valuable part of the day?	
Learning more about Wadsworth and all the divisions	10
Lab tours	6
Employee resources	2
What improvements can be made?	
Split up division talks with some breaks	5
Length of Event/Timing	2
Nothing	4
Other (Admin office tour, more info on EAP and benefits, etc.)	5
Additional feedback	
It was amazing, thank you	7
It was very informative, valuable, and enjoyable	6
None	4
Other (provide coffee and name tags)	2

Feedback from new employees that participated in the first Orientation Program highlighted the value in learning about the Center, the facility tour, and access to employee resources while suggesting changes to the overall structure of the time, including employee name tags, and adding an administration tour. In the second session, attendees noted fewer and more minor areas for improvement, which will be addressed in the third section.

Overall satisfaction for the program was rated ≥7/10 for all respondents across the two sessions, with 82% designating the program as a 9 or 10. Notably, the overall satisfaction ratings ≥9 increased from 75% to 91% in the second session relative to the first session based on improvements made in response to the feedback in Table 4.

### 3.3 Learning groups

**Figure 3:**
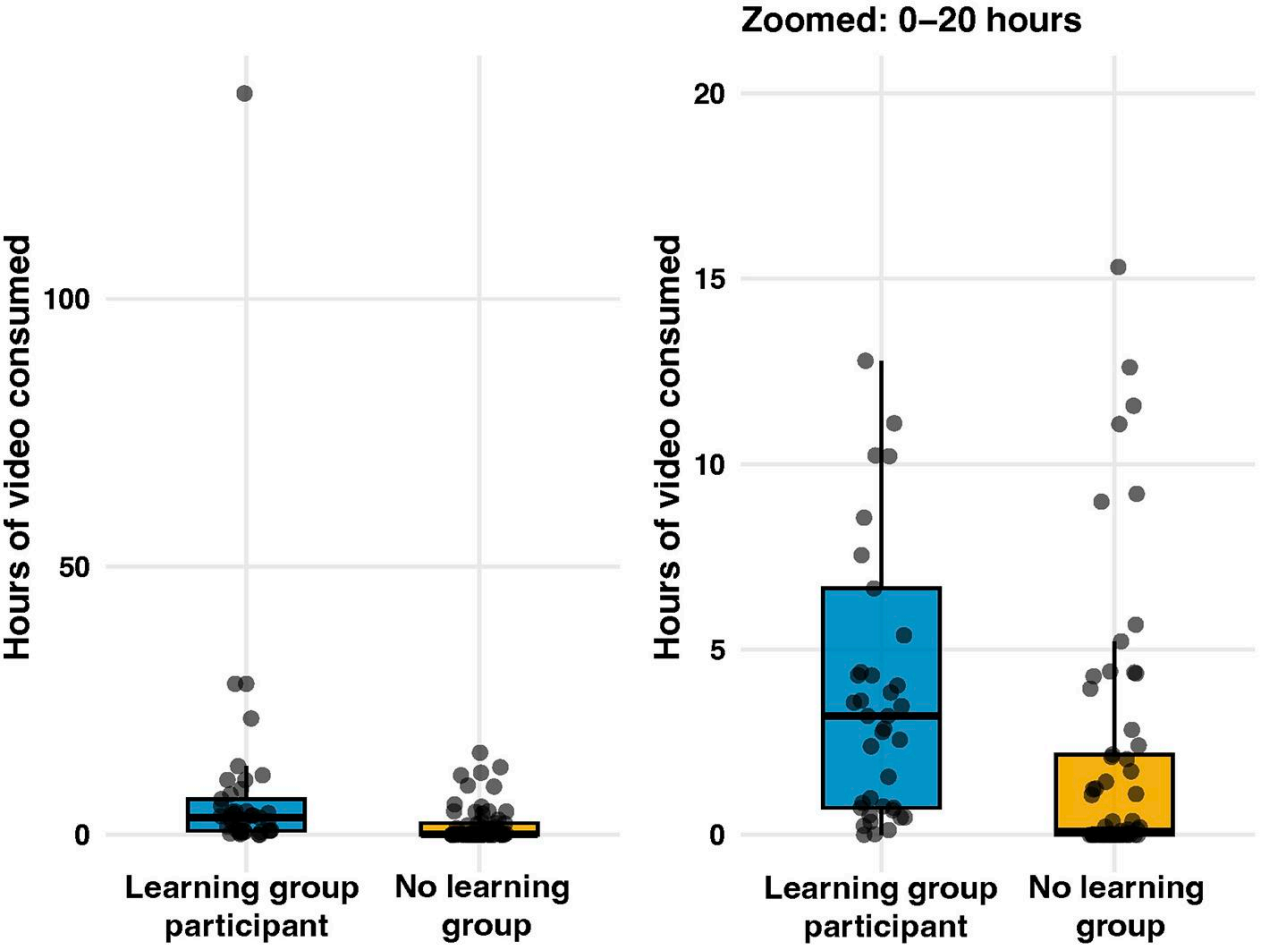
Overall number of hours of video consumed for users in at least one learning group relative to other users. Right panel: *y*-axis adjusted to show 0-20 h.

Platform data revealed striking differences in engagement. Over the three-month pilot period, the median number of hours spent on Udemy was 35 times higher among learning group participants (3.3 h, *n* = 36) compared to other users (0.1 h, *n* = 61, *p* < 0.0001 based on two-tailed Mann–Whitney U test) (Figure 3).

Survey feedback from 23 to 36 learning group participants further reinforced the added value of the learning group model and is summarized in Table 5. Seventy percent of respondents rated the groups as highly useful, while the remaining 30% rated them as neutral; no respondents gave a negative rating. When asked what aspects were most helpful, 61% specifically cited opportunities to learn from peers, underscoring the role of community and shared accountability. Suggestions for improvement included greater efforts to engage group members (five respondents) and dedicated time for participation (two respondents).

**Table 5:** Learning group survey data.

Rating (n responses; percent of total)	Representative statements
Learning Group was Very/Mostly Useful (16; 70%)	The group helps me understand the aspect of the course that are difficult to comprehend.
	It was an additional incentive to go through the class, do the exercises on time and be focused during the meeting.
	I learned more about useful resources for professional development.
	The R learning group has increased my awareness of the capabilities of R and how I can use them in my current role. Very open and receptive environment
	It was good to discuss management styles and get ideas from other leaders.
	The learning group provided accountability, support, and vision for how I could use my Udemy license.
	Forum to communicate with other R users
	Answered questioned I had on the subject.
Learning Group was Neutral Usefulness (7; 30%)	I haven't been able to attend many, but I do hope I can. The group collaboration part is what interested me in the first place.
	I didn't have the time to commit to learning a brand new skill but [the facilitator] was very knowledgeable and enthusiastic, answering questions outside of group meetings.
	Besides a presentation that I enjoyed. Not much
	It's been nice to connect with people from different departments who have similar interests and talk about them.

23/36 (64%) Learning group participants responded to the survey. Statements are direct quotes.

The learning group model evolved following the conclusion of the pilot based on community values:

1. The Python group continued using the original model before concluding in the Spring of 2025. It was rebooted at a larger scale from October 2025 – February 2026.
2. The Leadership/Management group concluded at the end of the pilot. The model was leveraged to launch a separate initiative targeting leadership development.
3. The AI group restructured as a workgroup focused on implementation strategies for the Center.
4. The R group shifted into a collaborative project space where members support each other's work and relaunched as a 10-week learning group session in January 2026.

### 3.4 CARE steering committee

In the initial 12-month period the Steering Committee was involved in completing 13 targeted objectives that aligned with the overall CARE program goals. The Committee led and/or supported six social events (e.g., a Center-wide picnic, volunteer work). A “Peer Training Program” was successfully developed and piloted with an initial session on artificial intelligence in life sciences. Databases of local academic contacts and staff interested in participating in academic engagement were developed, a process consistent with internal guidelines was established, and five of the outreach events noted in the section above were organized by the Steering Committee. Two partnerships were established with local organizations that focus on public-facing scientific speakers, and one public speaking training session was conducted to facilitate staff engagement.

**Figure 4:**
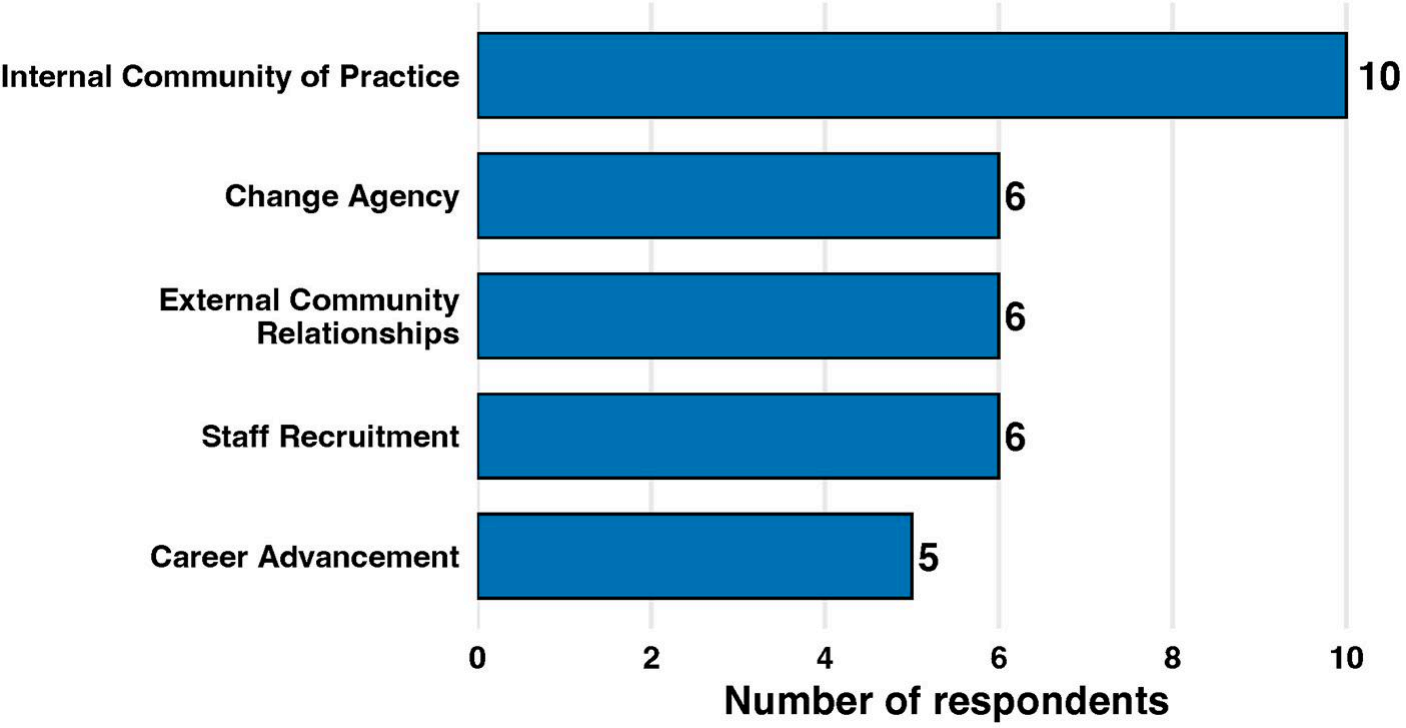
Thematic summary of steering committee member responses to the question “what does the WC CARE mission mean to you?”.

**Figure 5:**
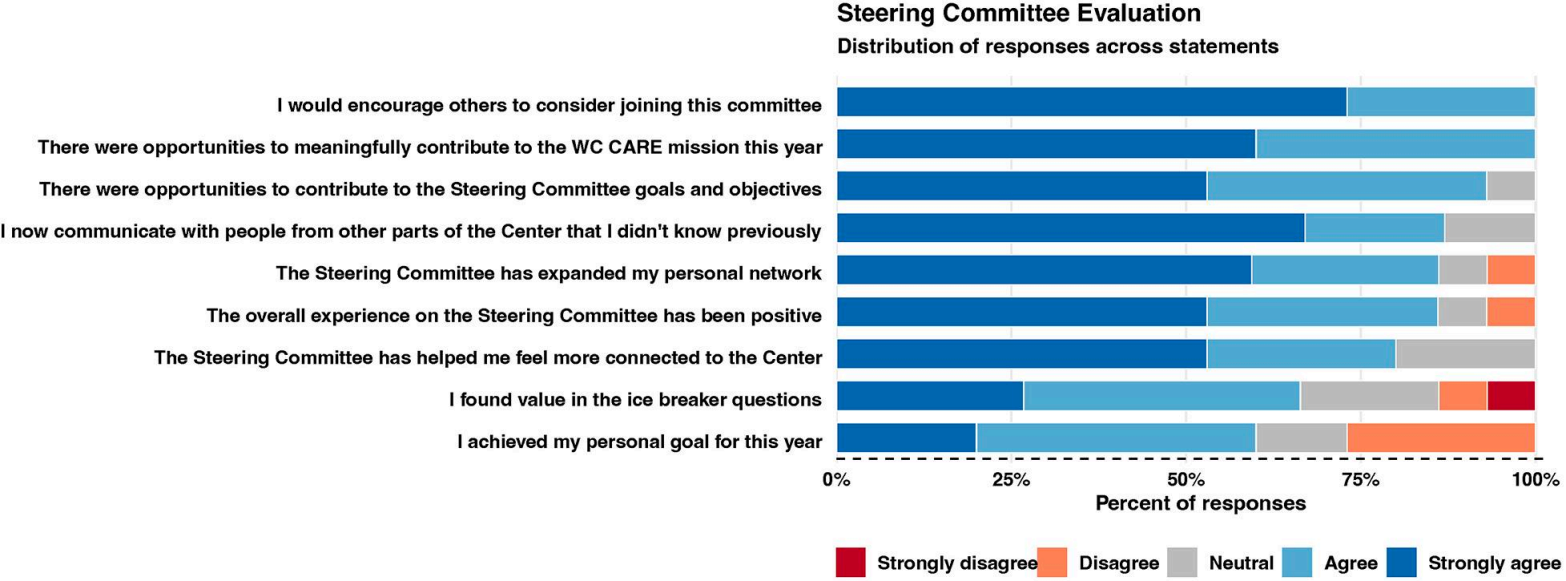
Likert summary of agreement for steering committee program evaluation.

The Steering Committee Evaluation was completed by 15/22 members (committee chair and retired member excluded). Responses are summarized in Figures 4, 5 for an open text question about the mission of CARE and the Likert responses, respectively.

Overwhelmingly, respondents felt the CARE mission was people centric – 14/15 responses included at least one aspect of the CARE mission (community, advancement, recruitment, engagement). Two-thirds wrote of working toward an internal community using words such as, “nurturing, equitable, productive, and connected” and “fulfilled and supported.”

One thematic element mentioned by six respondents that is not embedded into the program mission was change. The opportunity to “restore', “correct”, “influence factors that have impact”, and “take tangible steps” are in line with the 100% agreement that there were opportunities to meaningfully contribute to the WC CARE mission. Additionally, the ownership the steering committee members felt was highlighted in their responses to the question “What is the primary purpose of the Steering Committee?” where nearly half indicated they felt steering committee members were representatives of their laboratory, division or level. Two wrote of the steering committee bringing equity across levels and creating “One Wadsworth.”

At least 80% of respondents agreed or strongly agreed that there were opportunities to contribute to the steering committee/to the CARE mission, that the committee has helped them expand their personal network and feel more connected to the Center, that their overall experience was positive and that they would encourage others to join. The lowest agreement indicated 27% of respondents did not meet their personal goal for the year while 14% did not find value in ice-breaker questions. Additional feedback centered on improving communication and increasing opportunities to interact across groups.

## 4 Discussion

### 4.1 Academic engagement

Outreach to schools serves both as an effective career pipeline (9, 10) and a method of building connections with communities (11, 12). The overall breadth and reach of the academic engagement was a significant success of the CARE program as demonstrated by the quantitative information shown in the results section. In addition, most staff enjoyed the experiences with the students and were interested in continued participation. However, the staff feedback was also clear that the relatively rapid ramp up was too ambitious to be sustainable. Many individuals had challenges navigating through the various processes associated with travel, time logging, and clearance of materials. Additional support structures are needed to overcome these challenges.

### 4.2 Wadsworth Center orientation program

Standard employee orientation programs put a focus on relaying important information about institutional structure, culture, and routine procedures (13) and a thoughtfully designed orientation program is shown to increase the socialization, knowledge, skills and abilities of new employees (14). Participant learner outcomes and overall satisfaction based on feedback from participants indicates high effectiveness. However, logistical issues with scheduling across five sites (40%–50% attendance rate) and covering the breadth of information in a way that is accessible to all new staff remain significant challenges for the scope of the Wadsworth Center.

### 4.3 Learning groups

The learning and community-building benefits of group-based modalities – often referred to as learning groups or communities of practice – are well-documented in professional literature (15). Quantitative data on Udemy usage, combined with the largely positive participant feedback, are indicative of early successes for this learning model. Beyond individual skill-building, the continued activity of three of the four groups demonstrates how the initiative has evolved into a platform for collaboration, peer support, and cross-disciplinary engagement extending beyond the learning material itself.

At the same time, several challenges were noted, including:

1. Competing time commitments
2. Scheduling and location constraints
3. Varying levels of prior knowledge among group members
4. Technical barriers related to software or resources
5. Limited number of Udemy licenses

The learning group pilot provides a compelling demonstration of how structured, peer-supported learning can advance both professional development and institutional cohesion and addressing these challenges will be essential to scaling and sustaining the model.

### 4.4 CARE steering committee

Program level steering committees are common in cross-institution workforce development efforts (for example, the de Beaumont Foundation Common Workforce Agenda and the Association of Public Health Laboratories Workforce Development Committee) and empowering internal ‘workforce champions’ was a key success reported previously within the Centers for Disease Control and Prevention (8). The internal focus and self-governance of the CARE Steering Committee was a powerful catalyst for staff ownership and engagement that was reflected in how members related the mission to themselves. This initiative was the single most effective programmatic component for the success and long-term institutional viability of CARE. Challenges in this group included developing structure and scope, cross-group communication, and developing skills among the group leads.

### 4.5 Establishing institutional foundations for CARE sustainability

Each of the initiatives were designed for unique goals and to address the Center's priority needs of effective collaboration, technical competence, and community connections. In addition, they work synergistically to establish conditions for institutional sustainability:

1. Establish relational infrastructure.
2. Minimize activation barriers to engagement.
3. Engage staff in meaningful opportunities to contribute.

The Orientation Program established a system for new staff meet people while learning about the workplace, the learning groups to leverage peer expertise for skill development, academic engagement to work with colleagues and excite students about science and laboratory-based public health, and the Steering Committee to engage staff representatives in the process to address workforce and community engagement challenges. In each, a small group of individuals were generally responsible for coordinating to reduce the burden on the larger group of participants.

Table 6 defines successes and challenges with respect to establishing the conditions for institutional sustainability. Overall, CARE was very effective with establishing relational infrastructure and engaging staff with meaningful opportunities to contribute. The challenges for both were primarily logistical in nature and most have resolved as the program has developed. Minimizing activation barriers was where the most significant challenges emerged, particularly related to factors we have no control over. CARE's success in the first 18 months resulted from staff who found enough value in meaningfulness and relational benefits to overcome the many activation barriers; continued success will depend upon improving that cost-value equation.

**Table 6:** Assessment of successes and challenges from the CARE pilot towards establishing long-term institutional sustainability.

Conditions for Institutional Sustainability	Successes	Challenges
Establish relational infrastructure	1) Facilitating connections worked: positive feedback across all initiatives citing opportunities to meet others as a benefit. 2) Peer learning matters: 61% (learning group) and 55% (orientation) of respondents cited learning from others as a highlight.	1) Remote and hybrid structures limit relational connection: Logistical challenges with uniting people across five physically separated sites limits the practicality of frequent in-person activities. 2) New groups are dysfunctional: At the start, each of the pilot initiatives included some level of group dysfunction, lack of communication, personality clashes, and/or disparate goals. Most groups moved through this phase, but some did not.
Minimize activation barriers to engagement	1) Small groups are best for organizing: Planning/facilitating all of the activities reported in this work have been distributed across small group units. 2) Executive support is essential: Senior leadership and supervisors were encouraged to support staff to get involved in these initiatives.	1) Priorities do not always allow participation: time is still a major barrier for optional activities, based on feedback from learning groups and academic engagement participants. 2) Competition for attention: awareness of opportunities is still inconsistent despite all-staff emails, posting on internal news, and hanging up flyers. 3) Government processes remain barriers: constraints around paperwork requirements, and institutional/union policies have discouraged some staff from participating in academic engagement and some internal community building activities. 4) Supervisor support varies: Not all supervisors support staff involvement in extracurricular activities. 5) Travel between sites limits accessibility: In some cases, staff have to travel an extra hour to attend a mid-day event at another site. 6) Practical limitations must be considered: Feedback from the Steering Committee indicated desire to pursue specific activities that were not feasible given institutional constraints, number of Udemy licenses available limited how widely we could advertise for the learning groups.
Engage staff in meaningful opportunities to contribute	1) Staff found value in contributing to a higher purpose: feedback from academic engagement and the steering committee cited the larger purpose as a key highlight of participating. 2) Organizational units need common vision: the successful planning groups in all initiatives were united by a common goal. 3) Responding to real needs builds credibility: all of the initiatives described here were responses to feedback collected in our concurrent work.	1) Ambition needs to be rooted in feasibility: The Steering Committee initially sought to address more than what was feasible for the group given other job responsibilities. 2) Initial demand exceeded pilot scope: Participation levels were high across initiatives, but piloting before scaling resulted in more interest than there was capacity.

The collective assessments presented in this work include strong positive feedback around connecting with others, affecting change, making a difference and participating in useful learning opportunities. Approximately 191 individuals engaged in at least one activity associated with CARE, with 57 individuals who participated in at least two over the 18-month time frame.

### 4.6 Limitations

The nature of this work includes several limitations to interpretation. The initiatives described, and CARE, were pilot scale in length of time, scope, and number of participants. The surveys used were not validated instruments and we did not account for potential confounders such as volunteer participation or responder bias. The scope of CARE is within institution, results may not be representative of other organizations, and we do not have a comparison group. While the data suggest the initiatives are leading toward the longer-term objectives, the work described does not establish a causal link.

## 5 Conclusions/future directions

The CARE pilot initiatives reported on here provide strong evidence of early programmatic success and indicate reasonable adoption across the Wadsworth Center. Based on the lessons learned from the pilot initiatives, next steps include:

- Establishing a community of practice to strengthen academic engagement.
- Scaling up learning groups based on python, R, and artificial intelligence.
- Reformatting the leadership/management learning group into a more structured leadership academy.
- Investigating the potential of offering a ‘Re-Orientation’ to existing staff.
- Improving organizational support structures for the Steering Committee.

The work reported here has demonstrated the successes and challenges of implementing an integrated initiative in a public health laboratory setting. This model has shown relative success within the Wadsworth Center and can be applied in part or in whole in other scientific institutions that are seeking practical strategies for workforce development and community engagement in these fiscally constrained times.

Box **Key takeaways.**

• Academic engagement:
  ○ Initial baseline process and tracking of academic engagement established: 16 months, 88 staff, 28 events, 21 schools/programs, 1,030 students.
  ○ High support from staff: >95% of staff survey responses reflected positive experiences and willingness to attend future events.
  ○ Challenges: navigating official process and preparation time.
• Wadsworth Center Orientation Program:
  ○ High program success: 79% of participants were able to correctly answer program assessment questions.
  ○ Strong satisfaction with program: ratings ≥9 increased from 75% to 91% between the first and second sessions.
  ○ Challenges: logistical issues with scheduling across five physical sites and ensuring relevance/accessibility of information for new staff.
• Learning Groups:
  ○ 35x higher engagement: Learning group participants spent a median of 3.3 h on Udemy vs. 0.1 h for other users (*p* < 0.0001).
  ○ High perceived value: 70% of participants rated learning groups as highly useful; the rest were neutral.
  ○ Peer learning matters: 61% cited *learning from others* as the main benefit.
  ○ Barriers to address: Limited dedicated time.
• Steering Committee:
  ○ Initial term was productive: 13 objectives were completed.
  ○ Met program goals: ≥80% agreed the opportunity was meaningful and increased their connection with others and to the Center.
  ○ Engaged staff: Six members specifically cited the opportunity to affect positive change in their workplace.
  ○ Suggestions for improvements: increase flexibility and communication within the group.

## Data Availability

The raw data supporting the conclusions of this article will be made available by the authors, without undue reservation.
